# Upstairs, downstairs: conserved and divergent CLAVATA signalling in shoot meristem development and root symbioses

**DOI:** 10.1093/aob/mcae192

**Published:** 2024-11-01

**Authors:** Tiana E Scott, Alejandro Correa-Lozano, Eloise Foo

**Affiliations:** Discipline of Biological Sciences, School of Natural Sciences, University of Tasmania, Private Bag 55, Hobart, Tasmania, 7001, Australia; Discipline of Biological Sciences, School of Natural Sciences, University of Tasmania, Private Bag 55, Hobart, Tasmania, 7001, Australia; Discipline of Biological Sciences, School of Natural Sciences, University of Tasmania, Private Bag 55, Hobart, Tasmania, 7001, Australia

**Keywords:** Nodulation, mycorrhizae, autoregulation, CLE peptide, CLAVATA, *Pisum sativum*, shoot apical meristem

## Abstract

**Background:**

The CLV3/EMBRYO-SURROUNDING REGION (CLE) peptides control plant development and response to the environment. Key conserved roles include the regulation of shoot apical meristems and the long-distance control of root colonization by nutrient-acquiring microbes, including the widespread symbioses with arbuscular mycorrhizal fungi and nodulation with nitrogen-fixing bacteria in legumes. At least some signalling elements appear to operate across both processes but clear gaps in our understanding remain. In legumes, although CLE peptide signalling has been examined in detail in symbioses, the role of this pathway in shoot apical meristem (SAM) development is poorly understood.

**Scope:**

In this Research in Context, we review the literature to clarify the conserved and divergent elements of the CLAVATA-CLE peptide signalling pathways that control SAM development, mycorrhizal colonization and nodulation. We used novel pea mutants to determine the role of CLE signalling in regulating SAM development of a model legume, including interactions with temperature.

**Conclusions:**

We found that in pea, both genetic and environmental buffering of the CLE pathway influence SAM development. In pea, the CLAVATA2 (CLV2) CLE receptor-like protein and the unknown gene product encoded by the *K301* gene are required to limit SAM size and floral organ production under cool conditions. In contrast, the CLAVATA1 receptor-like kinase promotes SAM proliferation and appears to do so via a CLV2-independent pathway. In contrast, we found no role for the RDN1 enzyme, capable of arabinosylating CLE peptides, in SAM development. Future studies in other legumes are required to examine the role of other CLE peptide signalling elements in SAM control. Studies in non-vascular mycorrhizal hosts could explore if the control of symbioses is also an ancestral role for this signalling pathway.

## INTRODUCTION

The CLV3/EMBRYO-SURROUNDING REGION (CLE) peptides have emerged as key regulators controlling plant development and response to the environment (Narasimhan and Simon, 2022; Bashyal *et al.*, 2024). CLEs are a family of short, secreted peptides that interact with a range of leucine-rich repeat receptor-like kinases (LRR-RLK) to control shoot and root development and response to biotic and abiotic signals (Narasimhan and Simon, 2022; Bashyal *et al.*, 2024). Mature CLE peptides, consisting of 12–13 amino acids, are processed from longer pre-propeptides and are often activated post-translationally by glycosylation (Ohyama *et al.*, 2009). CLEs are often mobile, with some moving between nearby cells and others moving across the whole plant body to integrate responses (Roy and Muller, 2022). The specificities of CLE signalling pathways are the result of stimuli inducing the expression of unique CLE peptides that in turn interact with a range of downstream receptor complexes and unique signalling cascades.

**Fig. 1.**
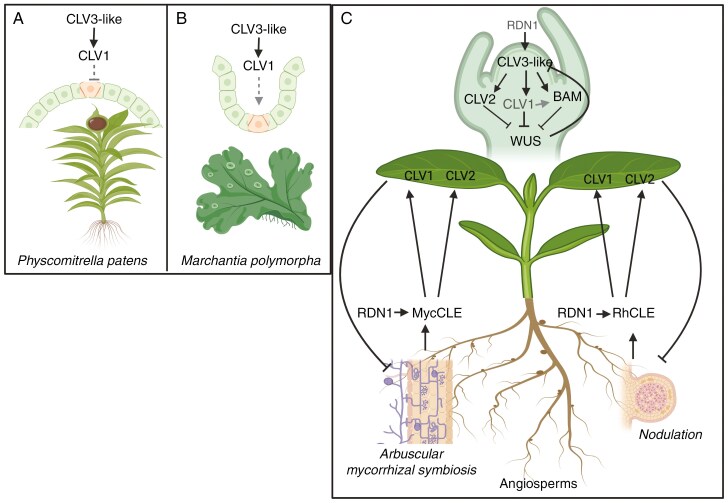
Simplified model of CLE peptide signalling control of shoot apical meristem function and symbioses in land plants to highlight common and divergent elements. (A) *Physcomitrella patens*, (B) *Marchantia polymorpha* and (C) evidence from studies across angiosperms. For A and B, apical cell shown in orange. For C, RDN1, CLV1 and arrow between CLV1 and BAM are shown in grey in shoot apex as there is inconsistency across species regarding their role based on mutant studies. Rh, rhizobial induced; Myc, mycorrhizal induced. Figure prepared using BioRender.

The founding member of the CLE family, CLAVATA3 (CLV3), is deeply conserved and signals through the CLAVATA1 (CLV1) RLK to control shoot apical meristem (SAM) cell proliferation and differentiation in land plants (reviewed by Hirakawa, 2022; Fig. 1). Another conserved role for CLE peptide signalling is the regulation of colonization by symbiotic microbes: arbuscular mycorrhizal (AM) fungi and, in legumes, colonization by nitrogen-fixing rhizobial bacteria leading to nodulation (Roy and Muller, 2022; Fig. 1). AM nutrient-acquiring symbiosis is ancient, emerging more than 450 Mya as plants moved onto land (Delaux *et al.*, 2015) and specific CLE peptides and a range of downstream receptors including CLV1 have been shown to negatively regulate AM colonization in several angiosperms (Wang *et al.*, 2018, 2021). Although the role of these elements in AM colonization of gymnosperms or bryophytes has not yet been examined, the fact that common CLE signalling elements also negatively regulate nodulation in legumes suggests an ancient role for CLE signalling in regulating symbioses.

In this review, we seek to clarify the conserved and divergent elements of the CLE peptide signalling pathways that control SAM development, autoregulation of mycorrhizal symbioses and autoregulation of nodulation (Fig. 1). It is important to point out that we do not provide a comprehensive review of the literature on CLE peptide control of SAM, which is beyond the scope of this paper and is covered in detail elsewhere (Hirakawa, 2022; Wang and Jiao, 2023). Instead, we identify a clear gap in our understanding of the role of CLE peptide signalling in SAM of legumes, that until now have been studied almost exclusively for the role of CLE signalling in symbioses. We carefully examine the shoot phenotypes of novel pea mutant combinations disrupted in key CLE peptide signalling genes and show that genetic and environmental buffering, hallmarks of CLV regulation of SAM in non-legumes, operate to control legume SAM development.

### CLE peptide signalling controls SAM development

In *Arabidopsis*, stem cell proliferation within the central zone of the SAM is tightly controlled through the CLV-WUS negative feedback loop (Fuchs and Lohmann, 2020). The CLV3 peptide is produced in the central zone and moves into the underlying organizing centre and is perceived by a combination of receptor complexes, including CLV1 (Deyoung and Clark, 2008; Guo *et al.*, 2010; Nimchuk *et al.*, 2015). CLV3 perception leads to the repression of *WUSCHEL* (*WUS*) expression in the organizing centre (Müller *et al.*, 2008; Song *et al.*, 2021), which encodes a homeobox transcription factor that maintains stem cell identity. WUS moves up into the central zone through plasmodesmata (Fuchs and Lohmann, 2020) and upregulates the expression of *CLV3* to increase stem cell abundance (Fig. 1C). Mutations in *CLV3*, *CLV1* and *WUS* lead to disrupted SAM development. Thus, the CLV-WUS negative feedback loop inhibits the over-proliferation and premature differentiation of stem cells in shoot apices controlling lateral organs such as flowers (Song *et al.*, 2021). Although application studies indicate post-translational arabinosylation does increase the activity of CLV3 (Ohyama *et al.*, 2009), the lack of a SAM phenotype in *Arabidopsis* mutants lacking all hydroxyproline O-arabinosyltransferase (HPAT) enzymes means the *in vivo* role for arabinosylation in CLV3 activity is still unclear (Song *et al.*, 2012; MacAlister *et al.*, 2016).

In addition to CLV1, there is evidence that the *At*CLV3 peptide is capable of binding with varying affinities to numerous combinations of homo- and hetero-receptor complexes to redundantly regulate stem cell abundance and differentiation through the repression of *WUS* (Müller *et al.*, 2008; Song *et al.*, 2021). This includes a receptor-like protein that lacks a kinase domain, *At*CLV2, the pseudokinase CORYNE (CRN), CLAVATA3 INSENSITIVE RECEPTOR KINASE1–4 (CIK1–4), RECEPTOR-LIKE PROTEIN KINASE (RPK2) and BARELY ANY MERISTEM 1–3 (BAM1–3) (Deyoung and Clark, 2008; Müller *et al.*, 2008; Guo *et al.*, 2010; Kinoshita *et al.*, 2010; Hu *et al.*, 2018). Putative receptor complexes that control CLV3 signalling in the SAM have been described based on phenotypes of double and triple mutants, and various protein–protein interaction assays are reviewed in detail elsewhere (Wang and Jiao, 2023). However, higher order mutant studies indicate CLV1 appears to act as a homo-dimer and plays the most prominent role in perceiving CLV3 in *Arabidopsis* (Clark *et al.*, 1993; Kayes and Clark, 1998; DeYoung *et al.*, 2006; Müller *et al.*, 2008; Kinoshita *et al.*, 2010; Hu *et al.*, 2018).

The CLV3–CLV1 signalling system operates to control shoot development across angiosperms including in rice, maize, petunia, groundcherry and tomato, with some important differences (Suzaki *et al.*, 2004, 2006; Xu *et al.*, 2015; Je *et al.*, 2016; Du *et al.*, 2022; Kwon *et al.*, 2022). Like *Arabidopsis*, disruption of tomato *SlCLV3*, CLV1-like *FASCIATED AND BRANCHED* (*FAB*) and *SlCLV2* leads to fasciated meristems and additional floral organs (Xu *et al.*, 2015). However, unlike mutant studies in *Arabidopsis*, disruption of the HPAT enzymes encoded by *FASCIATED INFLORESCENCE* (*FIN*) and *FAB2* in tomato also causes shoot fasciation, indicating that arabinosylation is important for suppression of SAM over-proliferation (Xu *et al.*, 2015; MacAlister *et al.*, 2016; Rodriguez-Leal *et al.*, 2019). However, the additive phenotype of *clv2 fin* and *fab fin* plants indicate there are multiple parallel pathways operating in tomato (Xu *et al.*, 2015; Wulf *et al.*, 2023). In rice, the CLV3 orthologue FLORAL ORGAN NUMBER2 (FON2) appears to signal through CLV1-like FON1 to restrict stem cells in floral meristems (Suzaki *et al.*, 2004, 2006). In maize, CLE peptide signalling controls stem cell proliferation in the inflorescence meristem (Du *et al.*, 2022). This includes several CLE-receptor complexes that suppress *ZmWUS*, including the CLV1-like receptor TASSEL DWARF (TD) that appears to interact with *Zm*CLE14, and the CLV2-like FASCIATED EAR2 (FEA2) that interacts with the closest homologue to CLV3, *Zm*CLE7, and the FEA-*Zm*CRN complex that interacts with the peptide ZmFON2-LIKE CLE PROTEIN (*Zm*FCP) (Chen *et al.*, 2021; Liu *et al.*, 2021; Du *et al.*, 2022). In addition to CLV3, many other CLE peptides also control SAM organization (reviewed by Selby and Jones, 2023) and this is touched on below when discussing genetic buffering. Parallel peptide signalling pathways also promote stem cell identity in *Arabidopsis*; for instance, the CLE40 peptide acts through BAM1 to promote stem cell identity in the organizing centre by promoting the expression of *WUS* (Schlegel *et al.*, 2021).

### Genetic and environmental buffering in CLE peptide signalling in SAM

SAM plasticity is regulated by different CLE peptides interacting with a range of receptor complexes, and it has recently emerged that genetic and environmental buffering of CLAVATA-CLE peptide signalling also occurs (Rodriguez-Leal *et al.*, 2019). This buffering process is also known as functional compensation, and the nature of this buffering differs across species and appears to have played a key role in the diversification of plant form (Rodriguez-Leal *et al.*, 2019; Kwon *et al.*, 2022). For example, growth temperature appears to influence CLV-WUS control of SAM and shoot organ development in several species (Wu *et al.*, 2023). In *Arabidopsis*, low temperature significantly enhances the shoot phenotypes of plants with mutations in key CLE signalling components (*clv3*, *clv1*, *clv2*, *cik1–4* and *crn* mutants; Jones *et al.*, 2021; John *et al.*, 2023). Careful analysis of mutants indicates the CLV2/CRN complex is required to maintain flower production under cool conditions by interacting with auxin (Jones *et al.*, 2021; John *et al.*, 2023). Similarly, low temperature enhanced the leaf and pod phenotypes of the soybean *clv1a* mutant (outlined in detail below; Mirzaei *et al.*, 2017). Recently, fruit formation in tomato was found to be disrupted by low temperature through the modification of *Sl*WUS mobility within the apex (Wu *et al.*, 2023). Overall, this suggests that at higher temperatures the CLE signalling system is buffered such that loss of individual components is less obvious, but under lower temperatures, this buffering is relaxed, adding an additional layer of complexity to enable environmental response.

Complex genetic buffering in CLV-WUS systems in SAMs is also observed across species. In *Arabidopsis*, loss of *CLV1* leads to upregulation of the expression of *BAM1* and *BAM2* in rib meristems, which, when combined with the extreme SAM phenotype of the *bam1 bam2 clv1* triple mutant, suggests active compensation of CLV1 loss by upregulation of *BAM* (Nimchuk *et al.*, 2015). In addition, mutation of *Sl*CLV3 in tomato results in upregulation of the expression of the paralogue *SlCLE9*, buffering the effect on SAM and fruit size (Rodriguez-Leal *et al.*, 2019). The true input of both *Sl*CLV3 and *Sl*CLV9 into SAM control are only exposed in the very severe phenotype of the *clv3 clv9* double mutants (Rodriguez-Leal *et al.*, 2019). A more detailed investigation into this interaction within the Solanaceae revealed that buffering differs markedly between species, with petunia and groundcherry exhibiting almost total compensation of CLV3 loss by CLE9 that might reflect an ancestral state, and the relaxion of this compensation in tomato was traced to modification of the CLV3 peptide and *cis*-regulatory elements (Kwon *et al.*, 2022). Similarly in maize, the expression of paralogues *ZmFCP1* and *ZmCLE15* appear to be upregulated to buffer the loss of the CLV3 orthologue *Zm*CLE7 (Rodriguez-Leal *et al.*, 2019; Liu *et al.*, 2021). In contrast to this active compensation, although multiple CLE peptides in addition to CLV3 appear to signal through CLV1/BAM in *Arabidopsis*, this compensation mechanism is passive (i.e. via genetic redundancy), with no additional CLE peptide gene expression upregulated in *clv3* mutants (Rodriguez-Leal *et al.*, 2019). Clearly this buffering means interpretation of single and higher order mutant phenotypes of CLE elements can be complex and can also be dependent on the growing environment.

### The CLV system is conserved in land plants

The conservation of core elements of the CLV signalling system in stem cell proliferation in moss and liverworts, without homologues in algae, suggests this pathway is a key feature of land plants (Whitewoods *et al.*, 2018; Hirakawa *et al.*, 2019, 2020). This includes key roles for CLV1/BAM-like RLK and CLE peptides (Fig. 1A, B). Unlike the negative role for CLV3 outlined in vascular plants, the CLV3 orthologue in *Marchantia polymorpha Mp*CLE2 signals through *Mp*CLV1 and *Mp*CIK receptor complexes to promote stem cells in gametophyte meristems, leading to promotion of dichotomous branching (Hirakawa *et al.*, 2019, 2020). In moss, two CLV1-like RLKs, *Pp*CLV1a and *Pp*CLV1b, and *Pp*RPK2 restrict stem cell number and are required for perception of ectopic CLV3 peptides in the gametophyte (Whitewoods *et al.*, 2018). Interestingly, HPAT enzymes have been shown to influence cell elongation in moss and *Arabidopsis*, consistent with a conserved role of these enzymes in modification of cell wall proteins (MacAlister *et al.*, 2016).

### The curious case of CLV1 in legume SAM development

Much of our understanding of the role of CLE peptide signalling in legumes relates to their function in symbioses, outlined below. However, given the most recent common ancestor of all land plants is believed to have used the CLV3–CLV1 pathway to control SAM, and that parallel CLE signalling also influences SAM development in a range of dicots, it seems likely that many of the core functions of this pathway would be retained in legumes. Indeed, *Lotus japonicus* transgenic plants with reduced expression of *LjCLV3* display a fasciated shoot apex and increased number of floral organs (Okamoto *et al.*, 2011). With the exception of soybean, which has undergone a recent whole genome duplication, many legumes contain a single CLV1 orthologue (see phylogeny in Schnabel *et al.*, 2005). However, curiously none of the *clv1* mutants present in legumes (pea, *Medicago*, *Lotus*) have been reported to display altered shoot development, with the exception of soybean (Searle *et al.*, 2003; Mirzaei *et al.*, 2017). In soybean, *GmCVL1a* and *GmNARK* are the closest homologues of *AtCLV1*, but only mutants disrupted in *GmCLV1a* displayed fasciated shoots, and altered leaves, flower number and pod development, while *Gmnark* mutants only display increased nodule number (Searle *et al.*, 2003; Mirzaei *et al.*, 2017).

Mutations in several other CLE signalling elements known to play a role in dicot SAM development are available in legumes. *Medicago* plants disrupted in the pseudokinase *MtCRN*, which acts as a coreceptor for CLV3 in *Arabidopsis*, displayed no change in shoot phenotype (Crook *et al.*, 2016). In contrast, disruption of legume orthologues of the *CLV2,* (*PsCLV2* and *LjCLV2*; Krusell *et al.*, 2011), and *AtRPK2*-like *LjKLAVIER* (*LjKLV*) gene, do display over-proliferation of shoot apices and increased number of floral structures (Oka‐Kira *et al.*, 2005; Miyazawa *et al.*, 2010). Like *Arabidopsis*, legume mutants disrupted in HPAT enzymes most closely related to *Sl*FIN (*Ps*RDN1, *Mt*RDN1, *Lj*PLENTY) and *bam* mutants have not been reported to display any difference in shoot development (Yoshida *et al.*, 2010; Schnabel *et al.*, 2011; Thomas and Frugoli, 2023). Given that no studies have explicitly set out to explore the role of CLE peptide signalling in legume shoot development, there are many unanswered questions. This includes the CLE-receptor complexes that control SAMs in legumes, including the role of CLV1, and the possibility of environmental, active or passive buffering of CLAVATA signalling in legume SAM control.

### CLE peptide signalling in symbioses

Symbioses with nutrient-acquiring microbes are beneficial to plants when nutrients are limited. Studies in both legumes and, more recently, non-legumes have established a key role of CLE peptide signalling in limiting the colonization of plant roots by symbiotic bacteria and fungi (Fig. 1C). Unlike the local cell-to-cell signalling outlined above for control of SAMs, autoregulation of symbioses occurs via a root–shoot–root feedback loop. This is a negative feedback loop with root colonization by symbionts inducing specific CLE peptides that move to the shoot and signal to the roots to suppress subsequent colonization by microbes (Roy and Muller, 2022). Autoregulation of AM fungi through this system appears to be ancestral, as CLE peptide signalling gene orthologues of *CLV1*, *CLV2* and HPAT enzymes control AM colonization in legumes and non-legumes (Wang *et al.*, 2018, 2021). Indeed, the conservation of this pathway is supported by the fact that split root studies in legumes indicate nodulation can systemically suppress AM and vice versa (Catford, 2003), and that legume mutants disrupted in *CLV1*, *CLV2* and *RDN1* (HPAT similar to *Sl*FIN) display increases in nodule number (Roy *et al.*, 2020) and an increase in the amount of root colonized by AM fungi (Solaiman *et al.*, 2000; Morandi *et al.*, 2009; Sakamoto and Nohara, 2009; Müller *et al.*, 2019; Karlo *et al.*, 2020). Some of the CLE signalling elements that limit symbioses overlap to some extent with nitrogen and phosphate signalling (reviewed by Roy and Muller, 2022; Valmas *et al.*, 2023) but this is beyond the scope of this review.

Although autoregulation of nodulation and mycorrhizal symbioses share the common signalling elements outlined above, the CLE peptides that regulate root colonization are unique to each symbiotic association, as are the downstream targets of CLE perception. Nodulation and in some cases Nod-factor application induces the expression of specific CLE peptides (*Medicago Mt*CLE12, *Mt*CLE13, *Mt*CLE35; pea *Ps*CLE12 and *Ps*CLE13; *Lotus Lj*CLE-RS1, *Lj*CLE-RS2, *Lj*CLE-RS3; soybean *Gm*RIC1, *Gm*RIC2; and bean *Pv*RIC1, *Pv*RIC2) (Lebedeva *et al.*, 2022; Roy and Muller, 2022). Evidence from application studies shows that at least some of these nodulation-activated CLE peptides require arabinosylation, and for some this occurs via the HPAT enzyme RDN1, although additional HPAT enzymes may be required for modification of other CLE peptides (Kassaw *et al.*, 2015, 2017; Imin *et al.*, 2018; Hastwell *et al.*, 2019; Yoro *et al.*, 2019*a*; Mens *et al.*, 2021). In *Lotus*, the nodulation-activated CLE glycopeptide CLE-RS2 moves through the phloem to the shoot where perception via the CLV1-like HAR1 takes place (Okamoto *et al.*, 2013). Grafting studies with receptor mutants, and the fact that root overexpression of nodulation-activated peptides require CLV1, CLV2, CRN and/or KLV in the shoot to suppress nodulation, suggest this long-distance transport and perception of CLE peptides in the shoot is conserved across species (Valmas *et al.*, 2023).

The precise receptor complexes that perceive CLE peptides to control nodulation in legumes is still unclear. Indirect evidence from BiFC (bimolecular fluorescence complementation) assays in *Nicotiana benthamiana* leaves have indicated no interaction between *Lotus japonicus* CLV1-like orthologue *Lj*HAR1 and *Lj*CLV2 (Krusell *et al.*, 2011), but in *Medicago* interactions were observed between the CLV1-like SUNN and *Mt*CLV2 when *Mt*CRN was included (Crook *et al.*, 2016), indicating a large complex with CLV1–CLV2–CRN occurs in these species, as has been proposed in *Arabidopsis* (Bleckmann *et al.*, 2009). Similar methods indicate *Lj*KLV can form homodimers and heteromeric complexes with *Lj*HAR1 (Miyazawa *et al.*, 2010). Consistent with CLV1 and CLV2 orthologues operating in complexes to control nodulation, double mutant *har1 clv2* plants were reported not to display an additive nodulation phenotype, although no quantitative data were presented (Krusell *et al.*, 2011). In *Medicago*, double mutant studies indicate RDN1 may act upstream of CLV1-like SUNN in *Mt*CLE12 perception (Kassaw *et al.*, 2017), although the additive nodulation phenotype of *Lotus* mutants disrupted in *CLV1*-like *HAR1* and *RDN1*-like *PLENTY* suggests these genes operate somewhat independently in this species (Yoro *et al.*, 2019*a*).

Perception of CLE peptides by CLV1 results in the downregulation of shoot-derived inhibitor miRNA2111, which is proposed to alleviate the repression of Kelch-repeat containing F-box protein genes *TOO MUCH LOVE1* and *2* (*TML1* and *TML2*) expression (Takahara *et al.*, 2013; Tsikou *et al.*, 2018; Zhang *et al.*, 2021). However, recent RNA sequencing studies showed that there was little change in the dynamics of *TML1* and *TML2* expression following inoculation in *sunn* and *rdn1* mutants compared to the wild-type, which suggests this pathway may be more complex (Schnabel *et al.*, 2023). There is also evidence for a role of shoot-derived cytokinin downstream of CLE perception in the shoot, but this has only been examined in *Lotus* (Sasaki *et al.*, 2014). The downstream targets of CLE peptide signalling in the root are still unclear but may include interactions with NIN, a transcription factor important in nodulation (Yoro *et al.*, 2019*b*), and downregulation of genes required for plant perception of rhizobia-produced Nod factor signals that are key to infection (Gautrat *et al.*, 2019). It is also interesting to note that like in SAM development, it is likely that parallel pathways control nodule number, as indicated by retention of some autoregulation responses in split root studies with *sunn* and *rdn1* mutants in *Medicago* (Kassaw *et al.*, 2015). In addition, a recent study suggests genetic compensation may operate between *BAM2* and *SUNN* in *Medicago* in nodulation control, suggesting BAM2 in roots may compensate for lack of SUNN activity (Thomas and Frugoli, 2023).

AM colonization is regulated in the legume *Medicago* by a unique set of AM-induced CLE peptides, including *Mt*CLE33 and *Mt*CLE53 (Müller *et al.*, 2019; Karlo *et al.*, 2020) and related CLE peptides *Sl*CLE10 and *Sl*CLE11 also control AM in the non-legume tomato (Wulf *et al.*, 2023). In *Medicago*, suppression of AM colonization by *Mt*CLE33 requires the arabinosylation enzyme *Mt*RDN1 and signals via the CLV1 orthologue *Mt*SUNN, although the role of other CLE signalling elements in this response (CLV2, CRN, KLV) has yet to be tested (Müller *et al.*, 2019; Karlo *et al.*, 2020). In tomato, *Sl*CLE11 action requires RDN1-orthologue FIN but does not appear to act via CLV1-like FAB or *Sl*CLV2, indicating other receptors must play a role (Wang *et al.*, 2021; Wulf *et al.*, 2023). The additive influence of mutations in *Sl*CLV2 and FIN on mycorrhizal colonization also support the notion of multiple parallel CLE pathways suppressing mycorrhizal colonization in tomato (Wulf *et al.*, 2023). Like autoregulation of nodulation, the precise receptor complexes and extent of possible parallel CLE peptide pathways in autoregulation of mycorrhizal symbioses has yet to be fully uncovered. Furthermore, the downstream targets of CLE peptide perception that limit AM colonization have yet to be discovered, but recent evidence suggests that, unlike nodulation, it does not include suppression of *TML1* or *TML2* expression in the roots (Schnabel *et al.*, 2023).

In this paper we used pea mutants previously characterized for their role in symbioses to carefully examine how CLAVATA-CLE peptide signalling influences SAM development in this model legume. Given the strong influence of temperature on CLV-WUS control of SAMs in other species, we first examined the influence of day and night temperature on two super-nodulating mutants known to display altered shoot phenotypes, *Psclv2* and *K301* (mutated in an unknown gene). We then used the permissive conditions identified to explore the role of genetic buffering in SAM regulation in pea by examining the shoot phenotypes of other super-nodulation mutants that have not been reported to display altered shoot phenotypes under ambient conditions (*Psnark* and *Psrdn1*), as well as novel double mutants between *Psnark*, *Psclv2* and *Psrdn1*.

## MATERIALS AND METHODS

### Plant material and genotyping

Plant genotypes were *Psnark* (P88, formerly *sym29*, disrupted in *PsCLV1*), *Psclv2* (P64, formerly *sym28*) and *Psrdn1* (P79, formerly *nod3*) derived from the parental line ‘Frisson’ (Duc and Messager, 1989; Sagan, 1996; Krusell *et al.*, 2002, 2011; Schnabel *et al.*, 2011) and K301 (also known as *nod4*, disrupted in an unknown gene) derived from ‘Ramonsky’ (Sidorova *et al.*, 2005; Sinjushin and Gostimskii, 2008). Double mutants between *Psnark*, *Psclv2* and *Psrdn1* were selected in the F2 generations using RFLP markers.

Primers for PCR amplification of template are as follows; *PsRDN11* F: 5ʹ-GAGAATGAAAGATGATCCAGAGAC-3ʹ, R: 5ʹ-AAGCAAGCACAAAATCGCCT-3ʹ*; PsNARK* F: 5ʹ-GAAATTCCACCGAGTTTTGG-3ʹ, R: 5ʹ-CAATCCGGTGAGGTGATTCT-3ʹ; PsCLV2 F: 5ʹ-TGCAGGCTTTGTTCCTTTC-3ʹ, R: 5ʹ-ACCGGGAAATCTTCCATACC-5ʹ. Restriction digests were carried out using the following enzymes: BsaJI for *PsRDN1*, BsmAI for *PsNARK* and BtsCI for *PsCLV2*. The *Psrdn1-2* mutant on Frisson background contains a C > T mutation that results in proline 274 being replaced with serine.

### Growing conditions and shoot measurements

Plants were grown two per 2-L pots in a 1:1 mixture of dolerite chips and vermiculite topped with potting mix. To assess the effect of day/night temperature on SAM phenotypes, plants were grown in growth cabinets with an 18-h day, 150 µmol m^-2^ s^−1^ light at pot height at one of three temperature regimes (20 °C day/15 °C night; 20 °C day/10 °C night; 15 °C day/15 °C night). To examine the effect of double mutant combinations, plants were grown in the same cabinets at 15 °C day/15 °C night. At maturity (7–8 weeks after planting), the phenotype of the shoot apex was rated using a fasciation score (Supplementary Data Table S1).

## RESULTS

### Low temperature enhances shoot defects in pea autoregulation of nodulation mutants

**Fig. 2.**
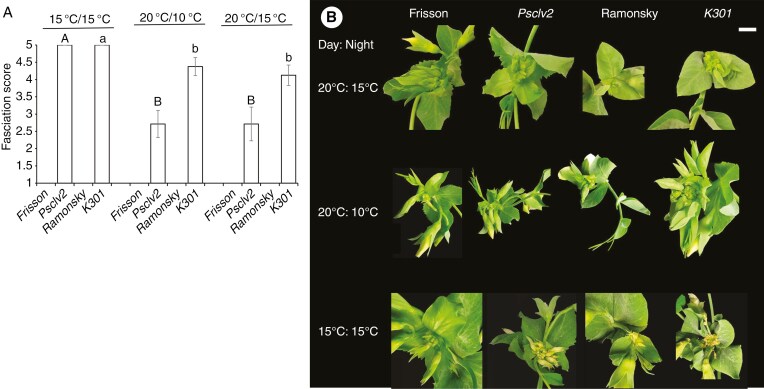
(A) Average fasciation score of shoot apex of mature pea plants (*Psclv2* vs Frisson; *K301* vs Ramonsky) grown under different temperature regimes (20 °C day/15 °C night; 20 °C day/10 °C night; 15 °C day/15 °C night). Values are mean ± standard error (*n* = 7–8); within a genotype letters indicate values that are significantly different (*P* < 0.05). (B) Typical shoot apex of mature pea plants. Scale bar = 1 cm.

We examined if lower temperatures influenced the severity of the SAM phenotype of two super-nodulating mutants of pea, *Psclv2* and *K301*, and wild-type lines, Frisson and Ramonsky respectively (Fig. 2). *Psclv2* mutants are disrupted in the conserved CLV2 receptor-like protein previously reported to display super-nodulation and fasciated shoots, including altered phyllotaxy, thickened and shortened stem, and additional floral organs coinciding with the onset of flowering (Krusell *et al.*, 2011). Under ambient glasshouse conditions the fasciation phenotype of *Psclv2* is highly variable (data not shown). *K301* mutant plants are disrupted in an unknown gene but, like *Psclv2*, displays super-nodulation and severe shoot fasciation at the onset of flowering (Sidorova *et al.*, 2005), along with duplication of leaves at some vegetative nodes (data not shown). Under all growth conditions both wild-type lines, Frisson and Ramonsky, displayed no shoot fasciation (Fig. 2). Under typical growth conditions for pea (20 °C day/15 °C night) and cooler nights (20 °C day/10 °C nights), *Psclv2* mutant plants and *K301* mutant plants displayed mild shoot fasciation. However, under 15 °C day/15 °C night, all *Psclv2* and *K301* mutants displaying extreme shoot fasciation.

### Shoot phenotypes of double mutants between Psnark, Psclv2 and Psrdn1

**Fig. 3.**
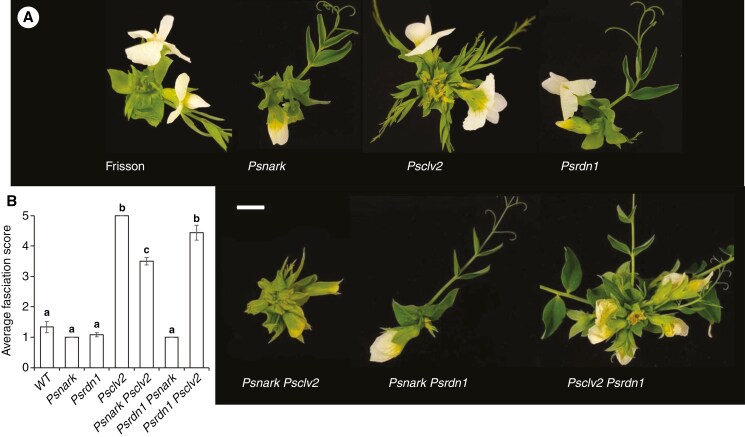
(A) Typical shoot apex of mature pea plants (Frisson, *Psnark*, *Psclv2*, *Psrdn1* and double mutants) grown under a 15 °C day/15 °C night. Scale bar = 1 cm. (B) Average fasciation score for each genotype; values are mean ± standard error (*n* = 5–6), and letters indicate values that are significantly different (*P* < 0.05).

We observed the shoot phenotypes of single mutant and double mutant plants disrupted in the CLV1-like gene *PsNARK*, *PsCLV2* and HPAT enzyme *PsRDN1* grown under 15 °C day/15 °C night to maximize any effect on shoot development and compared them to wild-type Frisson (Fig. 3). We scored the severity of the fasciation phenotype of individual plants using the fasciation score (Supplementary Data Table S1). No shoot fasciation was observed in wild-type, *Psnark*, *Psrdn1* or *Psnark rdn1* double mutant plants (Fig. 3). As observed previously (Fig. 2), all *Psclv2* plants displayed severe shoot fasciation under these growth conditions (Fig. 3). Strikingly, the severity of the shoot fasciation was significantly reduced in *Psnark clv2* double mutant plants compared to *Psclv2* mutants, although this was still significantly higher than in *Psnark* or wild-type plants. In contrast, *Psrdn1 clv2* mutants displayed no significant reduction in shoot fasciation compared to *Psclv2* single mutant plants, with all *Psrdn1 clv2* plants displaying severe fasciation.

## DISCUSSION

We sought to clarify the role of CLAVATA-CLE peptide signalling in SAM control in the model legume pea, including examining the role of CLV1, interactions with the environment and other elements of the CLE peptide signalling pathway, CLV2 and RDN1. We found that CLV1 does play a role in regulating SAM development of the model legume pea, in addition to the well-characterized role of this receptor in symbioses (Fig. 1C). This is a key finding as it addresses the implication in the literature that the CLAVATA-CLE peptide signalling system in SAM control is fundamentally different in legumes, and instead illustrates that legumes are another example of how this system is rewired in different land plant groups and species. Furthermore, we found no evidence supporting a role for RDN1 in SAM control in pea, which indicates the role for this gene in SAM is so far restricted to tomato (Xu *et al.*, 2015). We also found that temperature has a profound influence on the expression of SAM phenotypes of CLE signalling elements, consistent with the findings in other angiosperms (Jones *et al.*, 2021).

Interestingly, unlike a negative role in restricting SAM size that has been found in many angiosperms and moss (Fig. 1), we found that CLV1 in pea promotes SAM development. In the *Psclv2* mutant background, the CLV1-like *Ps*NARK positively influenced SAM development in pea, possibly by genetic buffering. This process may also explain why pea mutant plants disrupted in the *CLV1* gene, *Psnark*, do not display SAM over-proliferation (Fig. 3), and this is consistent with other *clv1* mutants in legumes that contain only one *CLV1* orthologue (Schnabel *et al.*, 2005). This might be due to active or passive compensation of other conserved elements of CLV-WUS pathway. A possible candidate for other CLE peptide signalling elements that might compensate for the loss of *Ps*NARK are CLV1-paralogues such as BAM proteins. For example, in *Arabidopsis*, *clv1* mutants display upregulation of the expression of *BAM1* and *BAM2* in rib meristems (Nimchuk *et al.*, 2015; Fig. 1C). A recent study in *Medicago* examined the expression of genes in shoot and roots of *sunn* (*clv1*-like mutant) and *rdn1* compared to wild-type in sterile conditions and after inoculation with bacteria (Schnabel *et al.*, 2023). No CLE peptide-related genes were reported to be upregulated in *sunn* mutant shoots (Schnabel *et al.*, 2023), although these samples were whole shoot tissue and genetic buffering is likely to occur specifically in SAM tissue. Buffering has not been explored in the CLAVATA-CLE peptide signalling control of symbioses, but it is interesting to note that several CLE peptides were ectopically upregulated in the roots of *sunn* and *rdn1* mutant plants compared to wild-type (Schnabel *et al.*, 2023). The fact that *Psnark clv2* double mutants actually displayed a reduction in shoot fasciation compared to *Psclv2* mutants suggests this compensation does not act via *Ps*CLV2. A somewhat independent role for *Ps*NARK and *Ps*CLV2 in SAM control is consistent with studies in *Arabidopsis* and tomato that suggest these proteins do not act as heterodimers to perceive CLE peptides (e.g. Guo *et al.*, 2010; Xu *et al.*, 2015). Loss of *PsRDN1* did not influence the severity of the SAM phenotype of *Psclv2* mutants, also consistent with *Ps*RDN1 not being required to activate CLE peptides perceived via CLV2 to suppress SAM over-proliferation in pea.

Characterization of the temperature response indicates environmental buffering may occur in SAM control of the model legume pea. As has previously been observed in *Arabidopsis*, tomato and soybean mutants with enlarged SAM and/or other shoot over-proliferation phenotypes, lower temperatures also enhanced SAM over-proliferation in pea mutants *Psclv2* and *K301* (Fig. 2). This suggests that under lower temperatures the loss of an individual component of peptide signalling, like *PsCLV2*, is not able to be compensated for by other paralogues or interacting elements. Indeed, increased severity of *Psclv2* phenotypes in low temperatures has also been reported to occur in *Arabidopsis* (Jones *et al.*, 2021). Although *K301* is disrupted in an as yet unidentified protein, the fact that the loss of this gene leads to SAM over-proliferation that is also under temperature control is consistent with this gene also playing a key role in SAM control.

Our results suggest that complex environmental and genetic buffering may occur in the CLE peptide signalling control of SAM development of pea. A somewhat different role for CLV1 in SAM development of the model legume pea is consistent with the diversity of CLAVATA-CLE peptide signalling buffering observed across species (e.g. Rodriguez-Leal *et al.*, 2019; Kwon *et al.*, 2022). Future studies should address the clear gaps in our understanding of how legumes such as pea use common and divergent CLE peptide signalling elements to control SAM and also limit symbioses. This includes further exploration of the role of CLE peptide signalling elements in SAM control in legumes using single and higher order mutants across legume species (e.g. KLAVIER, CRN) and examination of genetic buffering in each developmental process. Beyond legumes, given the deep conservation of at least some CLE signalling elements in both SAM control and symbiosis in angiosperms, it would be fascinating to examine if this shared signalling pathway is ancestral by examining CLE peptide signalling in control of symbioses in mycorrhizal bryophyte hosts such as *Marchantia palaeace*.

## SUPPLEMENTARY DATA

Supplementary data are available at *Annals of Botany* online and consist of the following.

Table S1. Fasciation score used to categorize shoot phenotypes of pea plants.

mcae192_suppl_Supplementary_Table_S1

## References

[CIT0001] Bashyal S, Gautam CK, Muller LM. 2024. CLAVATA signaling in plant–environment interactions. Plant Physiology 194: 1336–1357. 37930810 10.1093/plphys/kiad591 PMC10904329

[CIT0002] Bleckmann A, Weidtkamp-Peters S, Seidel CAM, Simon R. 2009. Stem cell signaling in arabidopsis requires CRN to localize CLV2 to the plasma membrane. Plant Physiology 152: 166–176. 19933383 10.1104/pp.109.149930 PMC2799354

[CIT0003] Catford JG, Staehelin C, Lerat S, Piché Y, Vierheilig H. 2003. Suppression of arbuscular mycorrhizal colonization and nodulation in split-root systems of alfalfa after pre-inoculation and treatment with Nod factors. Journal of Experimental Botany 54: 1481–1487. 12709494 10.1093/jxb/erg156

[CIT0004] Chen Z, Li W, Gaines C, Buck A, Galli M, Gallavotti A. 2021. Structural variation at the maize WUSCHEL1 locus alters stem cell organization in inflorescences. Nature Communications 12: 2378. 10.1038/s41467-021-22699-8 PMC8062686 33888716

[CIT0005] Clark SE, Running MP, Meyerowitz EM. 1993. CLAVATA1, a regulator of meristem and flower development in Arabidopsis. Development 119: 397–418. 8287795 10.1242/dev.119.2.397

[CIT0006] Crook AD, Schnabel EL, Frugoli JA. 2016. The systemic nodule number regulation kinase SUNN in *Medicago truncatula* interacts with MtCLV2 and MtCRN. The Plant Journal: for Cell and Molecular Biology 88: 108–119. 27296908 10.1111/tpj.13234

[CIT0007] Delaux P-M, Radhakrishnan GV, Jayaraman D, et al 2015. Algal ancestor of land plants was preadapted for symbiosis. Proceedings of the National Academy of Sciences of the United States of America 112: 13390–13395. 26438870 10.1073/pnas.1515426112 PMC4629359

[CIT0009] Deyoung BJ, Clark SE. 2008. BAM receptors regulate stem cell specification and organ development through complex interactions with CLAVATA signaling. Genetics 180: 895–904. 18780746 10.1534/genetics.108.091108 PMC2567389

[CIT0008] DeYoung BJ, Bickle KL, Schrage KJ, Muskett P, Patel K, Clark SE. 2006. The CLAVATA1-related BAM1, BAM2 and BAM3 receptor kinase-like proteins are required for meristem function in Arabidopsis. The Plant Journal: for Cell and Molecular Biology 45: 1–16. 16367950 10.1111/j.1365-313X.2005.02592.x

[CIT0010] Du Y, Wu B, Xing Y, Zhang Z. 2022. Conservation and divergence: regulatory networks underlying reproductive branching in rice and maize. Journal of Advanced Research 41: 179–190. 36328747 10.1016/j.jare.2022.01.012 PMC9637487

[CIT0011] Duc G, Messager A. 1989. Mutagenesis of pea (*Pisum sativum* L.) and the isolation of mutants for nodulation and nitrogen fixation. Plant Science 60: 207–213.

[CIT0012] Fuchs M, Lohmann JU. 2020. Aiming for the top: non-cell autonomous control of shoot stem cells in Arabidopsis. Journal of Plant Research 133: 297–309. 32146616 10.1007/s10265-020-01174-3 PMC7214502

[CIT0013] Gautrat P, Mortier V, Laffont C, et al 2019. Unraveling new molecular players involved in the autoregulation of nodulation in *Medicago truncatula*. Journal of Experimental Botany 70: 1407–1417. 30753553 10.1093/jxb/ery465 PMC6382332

[CIT0014] Guo Y, Han L, Hymes M, Denver R, Clark SE. 2010. CLAVATA2 forms a distinct CLE-binding receptor complex regulating Arabidopsis stem cell specification. Plant Journal 63: 889–900. 10.1111/j.1365-313X.2010.04295.x PMC2974754 20626648

[CIT0015] Hastwell AH, Corcilius L, Williams JT, Gresshoff PM, Payne RJ, Ferguson BJ. 2019. Triarabinosylation is required for nodulation-suppressive CLE peptides to systemically inhibit nodulation in *Pisum sativum*. Plant Cell and Environment 42: 188–197. 10.1111/pce.13325 29722016

[CIT0016] Hirakawa Y. 2022. Evolution of meristem zonation by CLE gene duplication in land plants. Nature Plants 8: 735–740. 35854003 10.1038/s41477-022-01199-7

[CIT0018] Hirakawa Y, Uchida N, Yamaguchi YL, et al 2019. Control of proliferation in the haploid meristem by CLE peptide signaling in *Marchantia polymorpha*. PLoS Genetics 15: e1007997. 30845139 10.1371/journal.pgen.1007997 PMC6424463

[CIT0017] Hirakawa Y, Fujimoto T, Ishida S, et al 2020. Induction of multichotomous branching by CLAVATA peptide in *Marchantia polymorpha*. Current Biology 30: 3833–3840.e4 e4. 32822612 10.1016/j.cub.2020.07.016

[CIT0019] Hu C, Zhu Y, Cui Y, et al 2018. A group of receptor kinases are essential for CLAVATA signalling to maintain stem cell homeostasis. Nature Plants 4: 205–211. 29581511 10.1038/s41477-018-0123-z

[CIT0020] Imin N, Patel N, Corcilius L, Payne RJ, Djordjevic MA. 2018. CLE peptide tri-arabinosylation and peptide domain sequence composition are essential for SUNN-dependent autoregulation of nodulation in *Medicago truncatula*. New Phytologist 218: 73–80. 29393515 10.1111/nph.15019

[CIT0021] Je BI, Gruel J, Lee YK, et al 2016. Signaling from maize organ primordia via FASCIATED EAR3 regulates stem cell proliferation and yield traits. Nature Genetics 48: 785–791. 27182966 10.1038/ng.3567

[CIT0022] John A, Smith ES, Jones DS, Soyars CL, Nimchuk ZL. 2023. A network of CLAVATA receptors buffers auxin-dependent meristem maintenance. Nature Plants 9: 1306–1317. 37550370 10.1038/s41477-023-01485-y PMC11070199

[CIT0023] Jones DS, John A, VanDerMolen KR, Nimchuk ZL. 2021. CLAVATA signaling ensures reproductive development in plants across thermal environments. Current Biology 31: 220–227.e5. 33157018 10.1016/j.cub.2020.10.008 PMC7855260

[CIT0024] Karlo M, Boschiero C, Landerslev KG, et al 2020. The CLE53-SUNN genetic pathway negatively regulates arbuscular mycorrhiza root colonization in *Medicago truncatula*. Journal of Experimental Botany 71: 4972–4984. 32309861 10.1093/jxb/eraa193 PMC7410177

[CIT0025] Kassaw T Jr., Bridges W, Frugoli J. 2015. Multiple Autoregulation of Nodulation (AON) signals identified through split root analysis of *Medicago truncatula sunn* and *rdn1* mutants. Plants 4: 209–224. 27135324 10.3390/plants4020209 PMC4844323

[CIT0026] Kassaw T, Schnabel SNE, Frugoli J. 2017. ROOT DETERMINED NODULATION1 is required for *M. truncatula* CLE12, but not CLE13, Peptide signaling through the SUNN receptor kinase. Plant Physiology 174: 2445–2456. 28592666 10.1104/pp.17.00278 PMC5543944

[CIT0027] Kayes JM, Clark SE. 1998. CLAVATA2, a regulator of meristem and organ development in Arabidopsis. Development 125: 3843–3851. 9729492 10.1242/dev.125.19.3843

[CIT0028] Kinoshita A, Betsuyaku S, Osakabe Y, et al 2010. RPK2 is an essential receptor-like kinase that transmits the CLV3 signal in Arabidopsis. Development 137: 3911–3920. 20978082 10.1242/dev.048199

[CIT0029] Krusell L, Madsen LH, Sato S, et al 2002. Shoot control of root development and nodulation is mediated by a receptor-like kinase. Nature 420: 422–426. 12442170 10.1038/nature01207

[CIT0030] Krusell L, Sato N, Fukuhara I, et al 2011. The Clavata2 genes of pea and *Lotus japonicus* affect autoregulation of nodulation. The Plant Journal: for Cell and Molecular Biology 65: 861–871. 21276104 10.1111/j.1365-313X.2010.04474.x

[CIT0031] Kwon CT, Tang L, Wang X, et al 2022. Dynamic evolution of small signalling peptide compensation in plant stem cell control. Nature Plants 8: 346–355. 35347264 10.1038/s41477-022-01118-w

[CIT0032] Lebedeva MA, Sadikova DS, Dobychkina DA, Zhukov VA, Lutova LA. 2022. CLAVATA3/EMBRYO SURROUNDING REGION genes involved in symbiotic nodulation in *Pisum sativum*. Agronomy 12: 2840.

[CIT0033] Liu L, Gallagher J, Arevalo ED, et al 2021. Enhancing grain-yield-related traits by CRISPR-Cas9 promoter editing of maize CLE genes. Nature Plants 7: 287–294. 33619356 10.1038/s41477-021-00858-5

[CIT0034] MacAlister CA, Ortiz-Ramírez C, Becker JD, Feijó JA, Lippman ZB. 2016. Hydroxyproline O-arabinosyltransferase mutants oppositely alter tip growth in *Arabidopsis thaliana* and *Physcomitrella patens*. The Plant Journal: for Cell and Molecular Biology 85: 193–208. 26577059 10.1111/tpj.13079 PMC4738400

[CIT0035] Mens C, Hastwell AH, Su H, Gresshoff PM, Mathesius U, Ferguson BJ. 2021. Characterisation of *Medicago truncatula* CLE34 and CLE35 in nitrate and rhizobia regulation of nodulation. The New Phytologist 229: 2525–2534. 33067828 10.1111/nph.17010

[CIT0036] Mirzaei S, Batley J, El-Mellouki T, et al 2017. Neodiversification of homeologous CLAVATA1-like receptor kinase genes in soybean leads to distinct developmental outcomes. Scientific Reports 7: 8878. 28827708 10.1038/s41598-017-08252-y PMC5566472

[CIT0037] Miyazawa H, Oka-Kira E, Sato N, et al 2010. The receptor-like kinase KLAVIER mediates systemic regulation of nodulation and non-symbiotic shoot development in *Lotus japonicus*. Development 137: 4317–4325. 21098572 10.1242/dev.058891

[CIT0038] Morandi D, le Signor C, Gianinazzi-Pearson V, Duc G. 2009. A *Medicago truncatula* mutant hyper-responsive to mycorrhiza and defective for nodulation. Mycorrhiza 19: 435–441. 19347373 10.1007/s00572-009-0242-5

[CIT0040] Müller R, Bleckmann A, Simon R. 2008. The receptor kinase CORYNE of Arabidopsis transmits the stem cell-limiting signal CLAVATA3 independently of CLAVATA1. Plant Cell 20: 934–946. 18381924 10.1105/tpc.107.057547 PMC2390746

[CIT0039] Müller LM, Flokova K, Schnabel E, et al 2019. A CLE-SUNN module regulates strigolactone content and fungal colonization in arbuscular mycorrhiza. Nature Plants 5: 933–939. 31477892 10.1038/s41477-019-0501-1

[CIT0041] Narasimhan M, Simon R. 2022. Spatial range, temporal span, and promiscuity of CLE-RLK signaling. Frontiers in Plant Science 13: 906087. 36092449 10.3389/fpls.2022.906087 PMC9459042

[CIT0042] Nimchuk ZL, Zhou Y, Tarr PT, Peterson BA, Meyerowitz EM. 2015. Plant stem cell maintenance by transcriptional cross-regulation of related receptor kinases. Development 142: 1043–1049. 25758219 10.1242/dev.119677 PMC4360179

[CIT0043] Ohyama K, Shinohara H, Ogawa-Ohnishi M, Matsubayashi Y. 2009. A glycopeptide regulating stem cell fate in *Arabidopsis thaliana*. Nature Chemical Biology 5: 578–580. 19525968 10.1038/nchembio.182

[CIT0044] Oka‐Kira E, Tateno K, Miura K, et al 2005. klavier (klv), A novel hypernodulation mutant of *Lotus japonicus* affected in vascular tissue organization and floral induction. Plant Journal 44: 505–515. 10.1111/j.1365-313X.2005.02543.x 16236159

[CIT0045] Okamoto S, Nakagawa T, Kawaguchi M. 2011. Expression and functional analysis of a CLV3-like gene in the model legume *Lotus japonicus*. Plant and Cell Physiology 52: 1211–1221. 21652543 10.1093/pcp/pcr071

[CIT0046] Okamoto S, Shinohara H, Mori T, Matsubayashi Y, Kawaguchi M. 2013. Root-derived CLE glycopeptides control nodulation by direct binding to HAR1 receptor kinase. Nature Communications 4: 2191. 10.1038/ncomms3191 23934307

[CIT0047] Rodriguez-Leal D, Xu C, Kwon C-T, et al 2019. Evolution of buffering in a genetic circuit controlling plant stem cell proliferation. Nature Genetics 51: 786–792. 30988512 10.1038/s41588-019-0389-8 PMC7274162

[CIT0049] Roy S, Muller LM. 2022. A rulebook for peptide control of legume–microbe endosymbioses. Trends in Plant Science 27: 870–889. 35246381 10.1016/j.tplants.2022.02.002

[CIT0048] Roy S, Liu W, Nandety RS, et al 2020. Celebrating 20 years of genetic discoveries in legume nodulation and symbiotic nitrogen fixation. The Plant Cell 32: 15–41. 31649123 10.1105/tpc.19.00279 PMC6961631

[CIT0050] Sagan M. 1996. Sym28 and Sym29, two new genes involved in regulation of nodulation in pea (*Pisum sativum* L.). Symbiosis 20: 229–245.

[CIT0051] Sakamoto K, Nohara Y. 2009. Soybean (*Glycine max* [L.] Merr.) shoots systemically control arbuscule formation in mycorrhizal symbiosis. Soil Science and Plant Nutrition 55: 252–257.

[CIT0052] Sasaki T, Suzaki T, Soyano T, Kojima M, Sakakibara H, Kawaguchi M. 2014. Shoot-derived cytokinins systemically regulate root nodulation. Nature Communications 5: 4983. 10.1038/ncomms5983 25236855

[CIT0053] Schlegel J, Denay G, Wink R, et al 2021. Control of Arabidopsis shoot stem cell homeostasis by two antagonistic CLE peptide signalling pathways. Elife 10: e70934. 34643181 10.7554/eLife.70934 PMC8594942

[CIT0054] Schnabel E, Journet EP, de Carvalho-Niebel F, Duc G, Frugoli J. 2005. The *Medicago truncatula* SUNN gene encodes a CLV1-like leucine-rich repeat receptor kinase that regulates nodule number and root length. Plant Molecular Biology 58: 809–822. 16240175 10.1007/s11103-005-8102-y

[CIT0056] Schnabel EL, Kassaw TK, Smith LS, et al 2011. The ROOT DETERMINED NODULATION1 gene regulates nodule number in roots of *Medicago truncatula* and defines a highly conserved, uncharacterized plant gene family. Plant Physiology 157: 328–340. 21742814 10.1104/pp.111.178756 PMC3165882

[CIT0055] Schnabel EL, Chavan SA, Gao Y, Poehlman WL, Feltus FA, Frugoli JA. 2023. A *Medicago truncatula* autoregulation of nodulation mutant transcriptome analysis reveals disruption of the SUNN pathway causes constitutive expression changes in some genes, but overall response to Rhizobia resembles wild-type, including induction of TML1 and TML2. Current Issues in Molecular Biology 45: 4612–4631. 37367042 10.3390/cimb45060293 PMC10297080

[CIT0057] Searle IR, Men AE, Laniya TS, et al 2003. Long-distance signaling in nodulation directed by a CLAVATA1-like receptor kinase. Science 299: 109–112. 12411574 10.1126/science.1077937

[CIT0058] Selby R, Jones DS. 2023. Complex peptide hormone signaling in plant stem cells. Current Opinion in Plant Biology 75: 102442. 37672866 10.1016/j.pbi.2023.102442

[CIT0059] Sidorova K, Shumny V, Glianenko M, Mischenko T, Vlasova EY, Gaeva T. 2005. Expression of the symbiotic nod4 pea gene against various genotypic backgrounds. Russian Journal of Genetics 41: 533–538. 15977818

[CIT0060] Sinjushin AA, Gostimskii SA. 2008. Genetic control of fasciation in pea (*Pisum sativum* L.). Russian Journal of Genetics 44: 702–708. 18727391

[CIT0061] Solaiman MZ, Senoo K, Kawaguchi M, et al 2000. Characterization of Mycorrhizas formed by *Glomus* sp. on roots of hypernodulating mutants of *Lotus japonicus*. Journal of Plant Research 113: 443–448.

[CIT0063] Song XF, Yu DL, Xu TT, Ren SC, Guo P, Liu CM. 2012. Contributions of individual amino acid residues to the endogenous CLV3 function in shoot apical meristem maintenance in Arabidopsis. Molecular Plant 5: 515–523. 22259020 10.1093/mp/ssr120

[CIT0062] Song X-F, Hou X-L, Liu C-M. 2021. CLE peptides: critical regulators for stem cell maintenance in plants. Planta 255: 5. 34841457 10.1007/s00425-021-03791-1

[CIT0064] Suzaki T, Sato M, Ashikari M, Miyoshi M, Nagato Y, Hirano H-Y. 2004. The gene FLORAL ORGAN NUMBER1 regulates floral meristem size in rice and encodes a leucine-rich repeat receptor kinase orthologous to Arabidopsis CLAVATA1. Development 131: 5649–5657. 15509765 10.1242/dev.01441

[CIT0065] Suzaki T, Toriba T, Fujimoto M, Tsutsumi N, Kitano H, Hirano H-Y. 2006. Conservation and diversification of meristem maintenance mechanism in *Oryza sativa*: function of the FLORAL ORGAN NUMBER2 gene. Plant and Cell Physiology 47: 1591–1602. 17056620 10.1093/pcp/pcl025

[CIT0066] Takahara M, Magori S, Soyano T, et al 2013. Too much love, a novel Kelch repeat-containing F-box protein, functions in the long-distance regulation of the legume–Rhizobium symbiosis. Plant and Cell Physiology 54: 433–447. 23390201 10.1093/pcp/pct022

[CIT0067] Thomas J, Frugoli J. 2023. Mutation of BAM2 rescues the sunn hypernodulation phenotype in *Medicago truncatula*, suggesting that a signaling pathway like CLV1/BAM in Arabidopsis affects nodule number. Frontiers in Plant Science 14: 1334190. 38273950 10.3389/fpls.2023.1334190 PMC10808729

[CIT0068] Tsikou D, Yan Z, Holt DB, et al 2018. Systemic control of legume susceptibility to rhizobial infection by a mobile microRNA. Science 362: 233–236. 30166437 10.1126/science.aat6907

[CIT0069] Valmas MI, Sexauer M, Markmann K, Tsikou D. 2023. Plants recruit peptides and micro RNAs to regulate nutrient acquisition from soil and symbiosis. Plants (Basel) 12: 187. 36616316 10.3390/plants12010187 PMC9824779

[CIT0072] Wang Y, Jiao Y. 2023. Cell signaling in the shoot apical meristem. Plant Physiology 193: 70–82. 37224874 10.1093/plphys/kiad309

[CIT0071] Wang CL, Reid JB, Foo E. 2018. The art of self-control - autoregulation of plant-microbe symbioses. Frontiers in Plant Science 9: 988. 30042780 10.3389/fpls.2018.00988 PMC6048281

[CIT0070] Wang C, Velandia K, Kwon CT, et al 2021. The role of CLAVATA signalling in the negative regulation of mycorrhizal colonization and nitrogen response of tomato. Journal of Experimental Botany 72: 1702–1713. 33186449 10.1093/jxb/eraa539

[CIT0073] Whitewoods CD, Cammarata J, Nemec Venza Z, et al 2018. CLAVATA was a genetic novelty for the morphological innovation of 3D growth in land plants. Current Biology 28: 2365–2376.e5. 30033333 10.1016/j.cub.2018.05.068 PMC6089843

[CIT0074] Wu J, Sun W, Sun C, et al 2023. Cold stress induces malformed tomato fruits by breaking the feedback loops of stem cell regulation in floral meristem. New Phytologist 237: 2268–2283. 36564973 10.1111/nph.18699

[CIT0075] Wulf K, Sun J, Wang C, et al 2023. The role of CLE peptides in the suppression of mycorrhizal colonization of tomato. Plant and Cell Physiology 65: 107–119. 10.1093/pcp/pcad124 PMC10799714 37874980

[CIT0076] Xu C, Liberatore KL, MacAlister CA, et al 2015. A cascade of arabinosyltransferases controls shoot meristem size in tomato. Nature Genetics 47: 784–792. 26005869 10.1038/ng.3309

[CIT0077] Yoro E, Nishida H, Ogawa-Ohnishi M, et al 2019a. PLENTY, a hydroxyproline O-arabinosyltransferase, negatively regulates root nodule symbiosis in *Lotus japonicus*. Journal of Experimental Botany 70: 507–517. 30351431 10.1093/jxb/ery364 PMC6322572

[CIT0078] Yoro E, Suzaki T, Kawaguchi M. 2019b. CLE-HAR1 systemic signaling and NIN-mediated local signaling suppress the increased rhizobial infection in the daphne mutant of *Lotus japonicus*. Molecular Plant-Microbe Interactions 33: 320–327. 31880983 10.1094/MPMI-08-19-0223-R

[CIT0079] Yoshida C, Funayama-Noguchi S, Kawaguchi M. 2010. plenty, a novel hypernodulation mutant in *Lotus japonicus*. Plant and Cell Physiology 51: 1425–1435. 20732950 10.1093/pcp/pcq115

[CIT0080] Zhang M, Su H, Gresshoff PM, Ferguson BJ. 2021. Shoot-derived miR2111 controls legume root and nodule development. Plant Cell and Environment 44: 1627–1641. 10.1111/pce.13992 33386621

