# Supplementary material for: Upstairs, downstairs: conserved and divergent CLAVATA signalling in shoot meristem development and root symbioses
Source: Ann Bot. 2024 Nov 1;136(5-6):1397–406. doi: 10.1093/aob/mcae192 (PMC12682826; doi:10.1093/aob/mcae192)
Supplement: mcae192_suppl_Supplementary_Table_S1 [file mcae192_suppl_supplementary_table_s1.docx]

Suppl Table 1 – Fasciation score used to categorise shoot phenotypes of pea plants.

| Shoot fasciation score | | Description | |
| --- | --- | --- | --- |
| 1 | \| No fasciation.  No unusual stem, apex or leaf phenotype. \| \| --- \| \|  \| | | 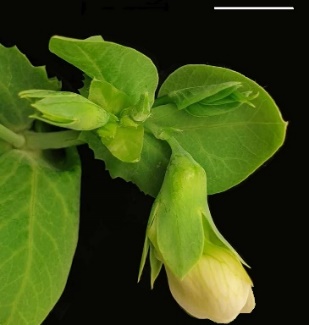 |
| 2 | Mild fasciation - apex slightly enlarged.  No unusual stem or leaf phenotypes. | | 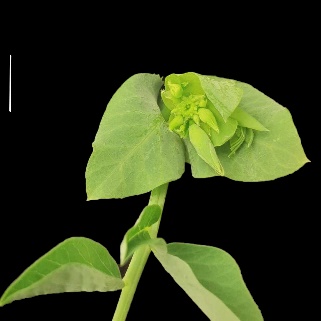 |
| 3 | Intermediate fasciation - apex enlarged with some additional leaf and floral organs.  Doubling of some petioles, commonly towards the apex. | | 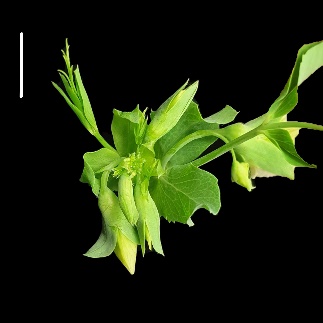 |
| 4 | Moderate fasciation - apex enlarged with additional leaf and floral organs.  Slight thickening of the stem, especially toward the apex, doubling of petioles, particularly toward the apex. | | 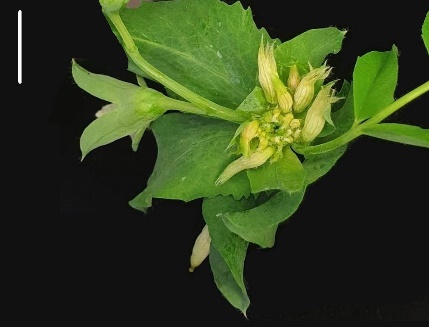 |
| 5 | Extreme fasciation - apex very enlarged with many additional leaf and floral organs.  Thickening of the stem, especially toward the apex, doubling of petioles. | | 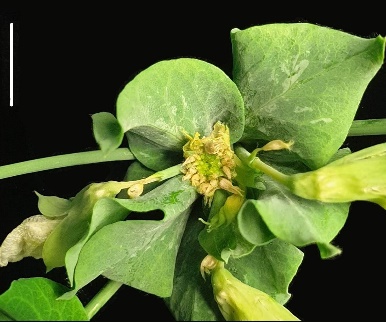 |

|  |
| --- |
